# Risk factors and predictive nomograms for early death of patients with advanced hepatocellular carcinoma: a large retrospective study based on the SEER database

**DOI:** 10.1186/s12876-022-02424-5

**Published:** 2022-07-19

**Authors:** Haidong Zhang, Xuanlong Du, Hui Dong, Wenjing Xu, Pengcheng Zhou, Shiwei Liu, Xin Qing, Yu Zhang, Meng Yang, Yewei Zhang

**Affiliations:** 1 Medical School, Southeast University, Nanjing, China; 2 Department of Ultrasound, State Key Laboratory of Complex Severe and Rare Diseases, Peking Union Medical College Hospital, Chinese Academy of Medical Sciences and Peking Union Medical College, Beijing, China; 3 Hepatopancreatobiliary Center, The Second Affiliated Hospital of Nanjing Medical University, Nanjing, China

**Keywords:** Hepatocellular carcinoma, SEER database, Early death, Risk factors, Nomogram

## Abstract

**Background:**

Hepatocellular carcinoma (HCC) is a kind of tumor with high invasiveness, and patients with advanced HCC have a higher risk of early death. The aim of the present study was to identify the risk factors of early death in patients with advanced HCC and establish predictive nomograms.

**Methods:**

Death that occurred within 3 months of initial diagnosis is defined as early death. Patients diagnosed with stage IV HCC between 2010 and 2015 were collected from the Surveillance, Epidemiology, and End Results database for model establishment and verification. Univariable and multivariable logistic regression analyses were used to identify the risk factors. Predictive nomograms were constructed and an internal validation was performed. Decision curve analysis (DCA) was used to verify the true clinical application value of the models.

**Results:**

Of 6603 patients (57% age > 60, 81% male, 70% white, 46% married), 21% and 79% had stage IVA and IVB, respectively. On the multivariable analyses, risk factors for early deaths in patients with stage IVA were age, tumor size, histological grade, alpha-fetoprotein (AFP), fibrosis score, tumor stage (T stage), surgery, radiotherapy, and chemotherapy, and that in stage IVB were age, histological grade, AFP, T stage, node stage (N stage), bone metastasis, lung metastasis, surgery, radiotherapy, and chemotherapy. The areas under the curves (AUCs) were 0.830 (95% CI 0.809–0.851) and 0.789 (95% CI 0.768–0.810) in stage IVA and IVB, respectively. Nomograms comprising risk factors with the concordance indexes (*C*-indexes) were 0.820 (95% CI 0.799–0.841) in stage IVA and 0.785 (95% CI 0.764–0.0.806) in stage IVB for internal validation (Bootstrapping, 1000re-samplings). The calibration plots of the nomograms show that the predicted early death was consistent with the actual value. The results of the DCA analysis show that the nomograms had a good clinical application.

**Conclusion:**

The nomograms can be beneficial for clinicians in identifying the risk factors for early death of patients with advanced HCC and predicting the probability of early death, so as to allow for individualized treatment plans to be accurately selected.

**Supplementary Information:**

The online version contains supplementary material available at 10.1186/s12876-022-02424-5.

## Background

Liver cancer is the fifth most frequent cancer in the world, ranking fourth in the incidence of cancer-related mortality [1, 2]. Hepatocellular carcinoma (HCC) accounts for over 80% of primary liver cancer and ranks second in cancer migration [3]. Due to the insidious symptoms and high metastatic potential thereof, over 30% of hepatocellular carcinoma patients already have extrahepatic metastases at the time of initial diagnosis [4], and the five-year relative survival rate is only 8.1% [5].

The prognosis of HCC has always been poor, and surgical treatment is usually the only treatment option [6]. However, only 5–15% of patients with early HCC have the opportunity to receive surgical treatment [7], which most commonly includes liver transplantation, liver resection, and radiofrequency ablation [8]. For patients who have lost the opportunity for surgery, studies have shown that compared with conservative treatment, transcatheter arterial chemoembolization (TACE) can increase the 2-year survival rate of patients with intermediate liver cancer by 23% [7]. At present, for patients with advanced HCC, sorafenib, an oral multi-kinase inhibitor, is the most accepted option around the world. However, in addition to the serious side effects and eventual drug resistance, the median survival time is only 12.3 months [9, 10]. Further, studies have shown that the survival rates among patients with advanced HCC at 1, 2, and 3 years were 29%, 16%, and 8% [11], and the median survival time was 5.3 months [12], indicating that advanced HCC patients were prone to early death. Therefore, early identification of risk factors for early death of advanced HCC patients and assessment of the incidence of early death will not only help clinicians discern high-risk patients in time, but also be conducive to reducing the pain and economic burden of patients. So far, there has been no research on the nomograms of early death for patients with advanced HCC. As such, developing nomograms to guide clinicians in identifying risk factors for early death of patients and implementing individualized treatment is of considerable significance.

In the present study, patients diagnosed with advanced HCC in the SEER database were included as the research objects to explore the risk factors of early death, and nomograms were constructed to evaluate the probability of early death (≤ 3 months).

## Methods

### Patients

**Fig. 1:**
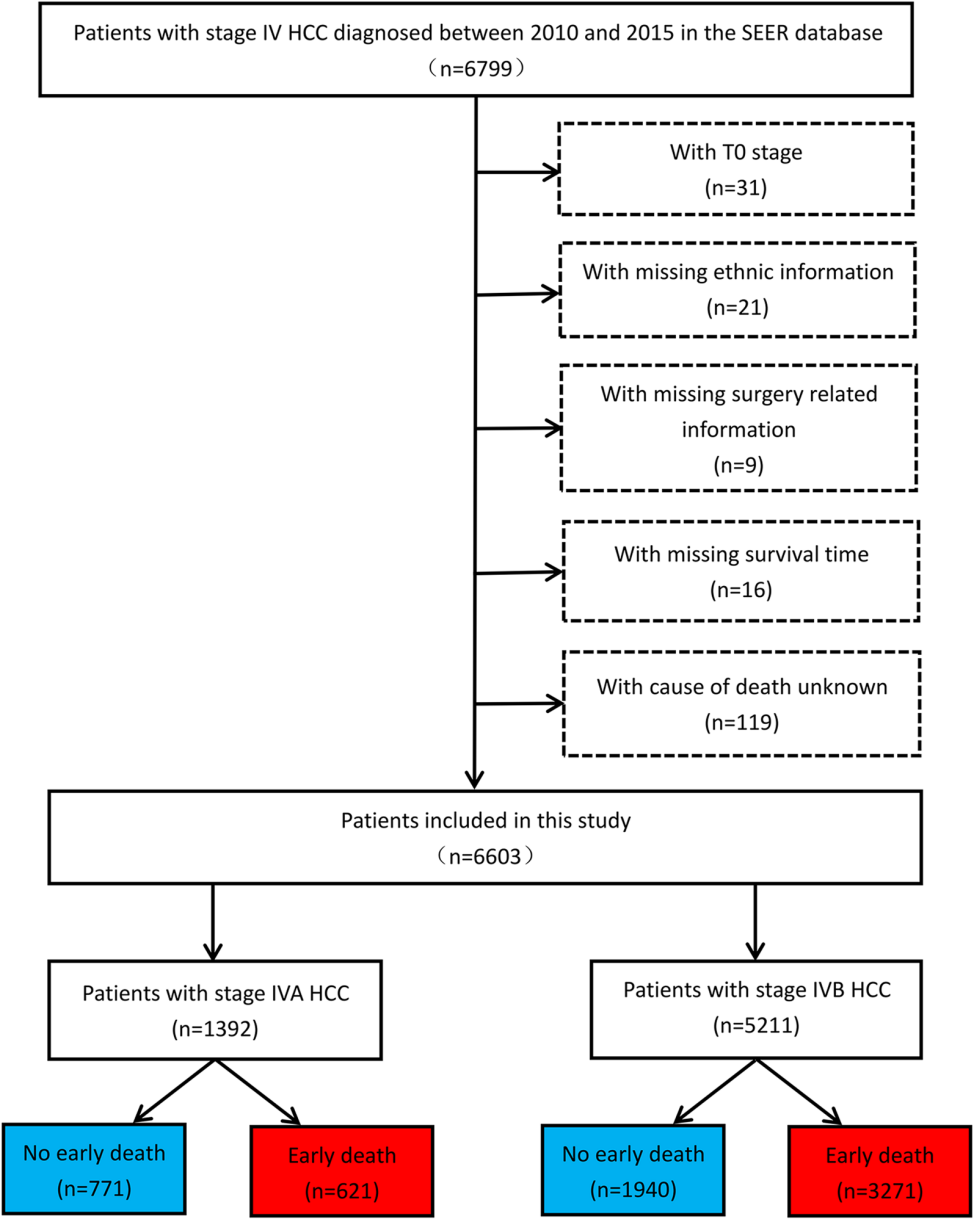
Patient selection flowchart. After selection criteria, 6603 patients were selected, of which 1392 were in stage IVA and 5211 were in stage IVB. 621 patients experienced early death in stage IVA, and 3271 patients in stage IVB suffered early death. Abbreviations: HCC, hepatocellular carcinoma

In the present study, SEER ∗ Stat (version 8.3.9.2) was used to collect all the relevant data, including patients' clinical information. The inclusion criteria were as follows: (1) patients with stage IV HCC registered between 2010 and 2015; (2) site code: C22.0; and (3) histological codes:8170/3-8175/3 [in the light of the International Classification of Tumor Diseases Third Edition (ICD-O-3)]. The exclusion criteria were as follows: (1) patients with T0 stage; (2) patients with missing ethnic information; (3) patients with missing surgery-related information; (4) patients with missing survival time; and (5) patients with the cause of death unknown. Figure 1 shows the patient selection flowchart. In consideration of the malignant degree and early metastasis performance of HCC as well as previous studies, early death was defined as death that occurs within 3 months after initial diagnosis [13, 14]. According to the latest American Joint Commission on Cancer (AJCC 8th) staging, patients with stage IVA HCC were defined as those with regional lymph node metastasis but without distant metastasis (IVA: T1–4; N1; M0); and patients with stage IVB HCC were defined as those with distant metastasis, whether with lymph node metastasis or not (IVB: T1–4; N0–1; M1) [15].

### Data collection

The information of patients with advanced HCC was extracted from the SEER database. The information included the following: (1) Baseline information including age, gender, race, and marital status; and (2) Clinical characteristics including tumor size, histological grade, AFP, fibrosis score (Ishak scoring system) [16], T stage, N stage, bone metastasis, brain metastasis, lung metastasis, surgery information, radiotherapy information, chemotherapy information, survival time, vital status, and cause of death.

### Statistical analysis

R software (Version 4.1.2; https://www.R-project.org) was used for all statistical analyses. The basic characteristics of the categorical variables in the patients were described using numbers and percentages (n, %), and were compared by means of the Chi-squared test. In the SEER data set, univariable and multivariable logistic regression analyses (Forward: LR) were used to identify variables that were significantly associated with early death of patients with advanced HCC. Two-sided *P*-values < 0.05 were considered statistically significant. Based on the statistically significant risk factors in the multivariable regression analysis, the R language “rms” package was used to develop predictive nomograms. The area under the receiver operating characteristic (ROC) curves (AUCs) were plotted to evaluate the discriminative performance of the nomograms [17]. Calibration curves were plotted to verify the accuracy and reliability of the nomograms [18]. Decision curves analysis (DCA) was performed to evaluate the applicability of the nomograms in clinical practice [19, 20]. Bootstrapping (1000 re-samplings) and cross-validation (k = 10) were performed for internal validation, comparing the *C*-indexes after bootstrapping between the verification model and the original data to measure the accuracy of the nomograms.

## Results

### Characteristics of patients

A total of 6799 patients with stage IV HCC in the SEER database were included in the present study. In accordance with the exclusion criteria, 6603 patients were selected, of which 1392 (Additional file 1: Table S1) were in stage IVA and 5211 (Additional file 2: Table S2) were in stage IVB. Among the patients, 44.6% of stage IVA patients and 62.8% of stage IVB patients experienced early death. In general, 57% of patients with advanced HCC were older than 60 years old and most were male (81%). Moreover, 70% of patients with advanced HCC were White and 16% were Black. The number of patients with tumors larger than 50 mm was over two times higher than those with tumors smaller than 50 mm. Over 65% of patients with advanced HCC were AFP positive. In stage IVB patients, 29.3% of patients had bone metastasis and 40.3% of patients had lung metastasis, but only 2.2% of patients had brain metastasis. Few patients received surgery (7.3% in stage IVA and 3.4% in stage IVB) and radiotherapy (9.3% in stage IVA and 16.0% in stage IVB), but a relatively large number of patients (over 35%) received chemotherapy, and patients receiving chemotherapy were less likely to suffer early death (21%). The characteristics of all patients are shown in Table 1.

**Table 1:** Characteristics with advanced HCC patients

Characteristics	AJCC stage IVA				AJCC stage IVB			
	Overall	Non early death	Early death	*P* (χ^2^)	Overall	Non early death	Early death	*P* (χ^2^)
	(N = 1392)	(N = 771)	(N = 621)		(N = 5211)	(N = 1940)	(N = 3271)	
*Age (%)*				0.007				0.005
< = 40	28 (2.0)	24 (3.1)	4 (0.6)		109 (2.1)	58 (3.0)	51 (1.6)	
41–60	554 (39.8)	313 (40.6)	241 (38.8)		2084 (40.0)	766 (39.5)	1318 (40.3)	
61–80	720 (51.7)	388 (50.3)	332 (53.5)		2571 (49.3)	959 (49.4)	1612 (49.3)	
> 80	90 (6.5)	46 (6.0)	44 (7.1)		447 (8.6)	157 (8.1)	290 (8.9)	
*Gender (%)*				0.437				0.538
Female	258 (18.5)	149 (19.3)	109 (17.6)		972 (18.7)	353 (18.2)	619 (18.9)	
Male	1134 (81.5)	622 (80.7)	512 (82.4)		4239 (81.3)	1587 (81.8)	2652 (81.1)	
*Race (%)*				0.599				0.129
White	992 (71.3)	543 (70.4)	449 (72.3)		3573 (68.6)	1363 (70.3)	2210 (67.6)	
Black	225 (16.2)	125 (16.2)	100 (16.1)		830 (15.9)	292 (15.1)	538 (16.4)	
Others*	175 (12.6)	103 (13.4)	72 (11.6)		808 (15.5)	285 (14.7)	523 (16.0)	
*Marital status (%)*				0.379				0.058
Unmarried	340 (24.4)	182 (23.6)	158 (25.4)		1344 (25.8)	474 (24.4)	870 (26.6)	
Married	670 (48.1)	387 (50.2)	283 (45.6)		2372 (45.5)	934 (48.1)	1438 (44.0)	
Divorced or separated	208 (14.9)	106 (13.7)	102 (16.4)		754 (14.5)	274 (14.1)	480 (14.7)	
Widowed	104 (7.5)	55 (7.1)	49 (7.9)		478 (9.2)	164 (8.5)	314 (9.6)	
Unknown	70 (5.0)	41 (5.3)	29 (4.7)		263 (5.0)	94 (4.8)	169 (5.2)	
*Tumor size (%)*				< 0.001				< 0.001
< = 20 mm	49 (3.5)	39 (5.1)	10 (1.6)		184 (3.5)	76 (3.9)	108 (3.3)	
21–50 mm	323 (23.2)	232 (30.1)	91 (14.7)		813 (15.6)	375 (19.3)	438 (13.4)	
51–100 mm	518 (37.2)	293 (38.0)	225 (36.2)		1534 (29.4)	621 (32.0)	913 (27.9)	
> 100 mm	275 (19.8)	121 (15.7)	154 (24.8)		1149 (22.0)	413 (21.3)	736 (22.5)	
Unknown	227 (16.3)	86 (11.2)	141 (22.7)		1531 (29.4)	455 (23.5)	1076 (32.9)	
*Histological grade (%)*				0.050				< 0.001
Grade I–II	284 (20.4)	169 (21.9)	115 (18.5)		889 (17.1)	438 (22.6)	451 (13.8)	
Grade III–IV	160 (11.5)	76 (9.9)	84 (13.5)		665 (12.8)	202 (10.4)	463 (14.2)	
Unknown	948 (68.1)	526 (68.2)	422 (68.0)		3657 (70.2)	1300 (67.0)	2357 (72.1)	
*AFP (%)*				< 0.001				< 0.001
Negative	188 (13.5)	132 (17.1)	56 (9.0)		624 (12.0)	290 (14.9)	334 (10.2)	
Positive	989 (71.0)	526 (68.2)	463 (74.6)		3383 (64.9)	1212 (62.5)	2171 (66.4)	
Unknown	215 (15.4)	113 (14.7)	102 (16.4)		1204 (23.1)	438 (22.6)	766 (23.4)	
*Fibrosis scores (%)*				0.016				< 0.001
Ishak 0–4	57 (4.1)	42 (5.4)	15 (2.4)		193 (3.7)	102 (5.3)	91 (2.8)	
Ishak 5–6	267 (19.2)	149 (19.3)	118 (19.0)		782 (15.0)	317 (16.3)	465 (14.2)	
Unknown	1068 (76.7)	580 (75.2)	488 (78.6)		4236 (81.3)	1521 (78.4)	2715 (83.0)	
*T stage (%)*				< 0.001				< 0.001
T1–2	497 (35.7)	346 (44.9)	151 (24.3)		1604 (30.8)	686 (35.4)	918 (28.1)	
T3–4	779 (56.0)	378 (49.0)	401 (64.6)		2462 (47.2)	888 (45.8)	1574 (48.1)	
TX	116 (8.3)	47 (6.1)	69 (11.1)		1145 (22.0)	366 (18.9)	779 (23.8)	
*N stage (%)*								< 0.001
N0	NA	NA	NA		3071 (58.9)	1208 (62.3)	1863 (57.0)	
N1	1392 (100)	N = 771 (100)	N = 621 (100)		1224 (23.5)	433 (22.3)	791 (24.2)	
NX	NA	NA	NA		916 (17.6)	299 (15.4)	617 (18.9)	
*Bone metastasis (%)*								< 0.001
No	NA	NA	NA		3441 (66.0)	1207 (62.2)	2234 (68.3)	
Yes	NA	NA	NA		1527 (29.3)	650 (33.5)	877 (26.8)	
Unknown	NA	NA	NA		243 (4.7)	83 (4.3)	160 (4.9)	
*Brain metastasis (%)*								0.450
No	NA	NA	NA		4801 (92.1)	1797 (92.6)	3004 (91.8)	
Yes	NA	NA	NA		114 (2.2)	43 (2.2)	71 (2.2)	
Unknown	NA	NA	NA		296 (5.7)	100 (5.2)	196 (6.0)	
*Lung metastasis (%)*								< 0.001
No	NA	NA	NA		2856 (54.8)	1289 (66.4)	1567 (47.9)	
Yes	NA	NA	NA		2098 (40.3)	555 (28.6)	1543 (47.2)	
Unknown	NA	NA	NA		257 (4.9)	96 (4.9)	161 (4.9)	
*Surgery (%)*				< 0.001				< 0.001
No	1291 (92.7)	677 (87.8)	614 (98.9)		5036 (96.6)	1798 (92.7)	3238 (99.0)	
Local tumor destruction	45 (3.2)	41 (5.3)	4 (0.6)		76 (1.5)	65 (3.4)	11 (0.3)	
Wedge resection	23 (1.7)	21 (2.7)	2 (0.3)		34 (0.7)	24 (1.2)	10 (0.3)	
Lobectomy	33 (2.4)	32 (4.2)	1 (0.2)		54 (1.0)	46 (2.4)	8 (0.2)	
Surgery but the specific operation unknown	NA	NA	NA		11 (0.2)	7 (0.4)	4 (0.1)	
*Radiotherapy (%)*				< 0.001				< 0.001
No/Unknown	1262 (90.7)	654 (84.8)	608 (97.9)		4377 (84.0)	1416 (73.0)	2961 (90.5)	
Yes	130 (9.3)	117 (15.2)	13 (2.1)		834 (16.0)	524 (27.0)	310 (9.5)	
*Chemotherapy (%)*				< 0.001				< 0.001
No/Unknown	757 (54.4)	283 (36.7)	474 (76.3)		3339 (64.1)	754 (38.9)	2585 (79.0)	
Yes	635 (45.6)	488 (63.3)	147 (23.7)		1872 (35.9)	1186 (61.1)	686 (21.0)	

Others*: American Indian/AK Native, Asian/Pacific Islander

*AJCC* American Joint Commission on Cancer, *HCC* hepatocellular carcinoma

### Risk factors analysis for early death

Univariable and multivariable logistic regression analyses were used to determine the risk factors for early death in advanced HCC. In stage IVA HCC patients, 9 risk factors were identified, including age (41–60/61–80/ > 80) [OR 4.097 (1.158–17.179)/4.444 (1.259–18.591)/ 2.675 (0.700–11.897), *P* < 0.05]; tumor size (21–50 mm/51–100 mm/ > 100 mm/Unknown) [OR 1.642 (0.746–3.880)/2.571 (1.173–6.054)/5.521 (2.443–13.370)/4.376 (1.897–10.795), *P* < 0.001]; histological grade (Grade I–II/Grade III–IV/Unknown) [OR 1.754 (1.079–2.869)/0.826 (0.590–1.156), *P* < 0.05]; AFP (Positive/Unknown) [OR 1.796 (1.191–2.728)/1.387 (0.844–2.289), *P* < 0.005]; fibrosis score (Ishak 0–4/Ishak 5–6) [OR 2.528 (1.174–5.650)/2.043 (0.993–4.369), *P* < 0.05]; T stage (T3–4/TX) [OR 2.061 (1.501–2.838)/1.714 (0.977–3.022), *P* < 0.001]; surgery (Local tumor destruction/Wedge resection/Lobectomy) [OR 0.187 (0.053–0.502)/0.083 (0.012–0.332)/0.020 (0.001–0.108), *P* < 0.001]; radiotherapy (Yes) [OR 0.080 (0.041–0.145), *P* < 0.001]; and chemotherapy (Yes) [OR 0.144 (0.109–0.190), *P* < 0.001]. In stage IVB HCC patients, 10 risk factors were identified including age (41–60/61–80/ > 80) [OR 1.728 (1.090–2.740)/1.804 (1.137–2.864)/1.440 (0.864–2.403), *P* < 0.05]; histological grade (Grade I–II/Grade III–IV/Unknown) [OR 2.178 (1.703–2.792)/1.400 (1.176–1.666), *P* < 0.001]; AFP (Positive/Unknown) [OR 1.462 (1.194–1.791)/1.240 (0.984–1.563), *P* < 0.001]; T stage (T3–4/TX) [OR 1.363 (1.171–1.588)/1.249 (1.030–1.516), *P* < 0.001]; N stage (N1/NX) [OR 1.210 (1.029–1.426)/0.994 (0.821–1.206), *P* < 0.05]; bone metastasis (Yes/Unknown) [OR 1.239 (1.050–1.463)/0.715 (0.487–1.057), *P* < 0.05]; lung metastasis (Yes/Unknown) [OR 2.195 (1.901–2.537)/1.214 (0.839–1.763), *P* < 0.001]; surgery (Local tumor destruction/Wedge resection/Lobectomy/Surgery but the specific operation unknown) [OR 0.139 (0.067–0.265)/0.204 (0.089–0.441)/0.077 (0.032–0.165)/0.649 (0.141–2.597), *P* < 0.001]; radiotherapy (Yes) [OR 0.342 (0.281–0.415), *P* < 0.001]; and chemotherapy (Yes) [OR 0.163 (0.142–0.187), *P* < 0.001]. Tables 2 and 3 show the results of univariable and multivariable logistic regression analysis.

**Table 2:** The univariable logistic regression analysis for analyzing the risk factors for early death of advanced HCC

Characteristics	AJCC stage IVA			AJCC stage IVB		
	OR	95% CI	*P*-value	OR	95% CI	*P*-value
*Age (%)*						
< = 40	Ref			Ref		
41–60	4.620	1.757–15.876	0.005	1.957	1.330–2.888	< 0.001
61–80	5.134	1.960–17.602	0.003	1.912	1.302–2.815	< 0.001
> 80	5.739	2.021–20.714	0.003	2.101	1.377–3.216	< 0.001
*Gender (%)*						
Female	Ref			Ref		
Male	1.125	0.857–1.481	0.398	0.953	0.824–1.101	0.514
*Race (%)*						
White	Ref			Ref		
Black	0.967	0.722–1.294	0.824	1.136	0.971–1.331	0.112
Others*	0.845	0.609–1.169	0.313	1.132	0.966–1.328	0.128
*Marital status (%)*						
Unmarried	Ref			Ref		
Married	0.842	0.648–1.096	0.200	0.839	0.730–0.964	0.013
Divorced or Separated	1.108	0.785–1.566	0.559	0.954	0.793–1.150	0.623
Widowed	1.026	0.660–1.593	0.908	1.043	0.839–1.301	0.706
Unknown	0.815	0.480–1.367	0.441	0.980	0.745–1.294	0.883
*Tumor size (%)*						
< = 20 mm	Ref			Ref		
21–50 mm	1.530	0.759–3.357	0.257	0.822	0.593–1.135	0.236
51–100 mm	2.995	1.520–6.462	0.003	1.035	0.756–1.409	0.830
> 100 mm	4.964	2.469–10.883	< 0.001	1.254	0.911–1.719	0.162
Unknown	6.394	3.146–14.148	< 0.001	1.664	1.213–2.273	0.001
*Histological grade (%)*						
Grade I–II	Ref			Ref		
Grade III–IV	1.624	1.100–2.404	0.015	2.226	1.804–2.753	< 0.001
Unknown	1.179	0.902–1.546	0.231	1.761	1.519–2.042	< 0.001
*AFP (%)*						
Negative	Ref			Ref		
Positive	2.075	1.489–2.923	< 0.001	1.555	1.309–1.847	< 0.001
Unknown	2.128	1.414–3.224	< 0.001	1.518	1.248–1.848	< 0.001
*Fibrosis scores (%)*						
Ishak 0–4	Ref			Ref		
Ishak 5–6	2.217	1.196–4.311	0.014	1.644	1.198–2.259	0.002
Unknown	2.356	1.320–4.435	0.005	2.001	1.498–2.676	< 0.001
*T stage (%)*						
T1–2	Ref			Ref		
T3–4	2.431	1.920–3.087	< 0.001	1.325	1.165–1.506	< 0.001
TX	3.364	2.224–5.129	< 0.001	1.591	1.358–1.865	< 0.001
*N stage (%)*						
N0	NA	NA	NA	Ref		
N1	NA	NA	NA	1.185	1.033–1.360	0.016
NX	NA	NA	NA	1.338	1.146–1.565	< 0.001
*Bone metastasis (%)*						
No	NA	NA	NA	Ref		
Yes	NA	NA	NA	0.729	0.644–0.825	< 0.001
Unknown	NA	NA	NA	1.042	0.794–1.375	0.771
*Brain metastasis (%)*						
No	NA	NA	NA	Ref		
Yes	NA	NA	NA	0.988	0.676–1.459	0.950
Unknown	NA	NA	NA	1.172	0.918–1.507	0.208
*Lung metastasis (%)*						
No	NA	NA	NA	Ref		
Yes	NA	NA	NA	2.287	2.025–2.584	< 0.001
Unknown	NA	NA	NA	1.380	1.063–1.800	0.017
*Surgery (%)*						
No	Ref			Ref		
Local tumor destruction	0.108	0.032–0.268	< 0.001	0.094	0.047–0.171	< 0.001
Wedge resection	0.105	0.017–0.360	0.002	0.231	0.105–0.471	< 0.001
Lobectomy	0.034	0.002–0.161	< 0.001	0.097	0.042–0.194	< 0.001
Surgery but the specific operation unknown	NA	NA	NA	0.317	0.083–1.052	0.067
*Radiotherapy (%)*						
No/Unknown	Ref			Ref		
Yes	0.120	0.064–0.206	< 0.001	0.283	0.242–0.330	< 0.001
*Chemotherapy (%)*						
No/Unknown	Ref			Ref		
Yes	0.180	0.142–0.227	< 0.001	0.169	0.149–0.191	< 0.001

Others*: American Indian/AK Native, Asian/Pacific Islander

*AJCC* American Joint Commission on Cancer, *HCC* hepatocellular carcinoma, *CI* confidence interval

**Table 3:** The multivariable logistic regression analysis for analyzing the risk factors for early death of advanced HCC

Characteristics	AJCC stage IVA			AJCC stage IVB		
	OR	95% CI	*P*-value	OR	95% CI	*P*-value
*Age (%)*						
< = 40	Ref			Ref		
41–60	4.097	1.158–17.179	0.037	1.728	1.090–2.740	0.020
61–80	4.444	1.259–18.591	0.027	1.804	1.137–2.864	0.012
> 80	2.675	0.700–11.897	0.167	1.440	0.864–2.403	0.161
*Marital status (%)*						
Unmarried	NA	NA	NA	Ref		
Married	NA	NA	NA	1.066	0.903–1.259	0.449
Divorced or Separated	NA	NA	NA	1.032	0.831–1.284	0.774
Widowed	NA	NA	NA	1.038	0.796–1.358	0.782
Unknown	NA	NA	NA	1.004	0.732–1.384	0.978
*Tumor size (%)*						
< = 20 mm	Ref			NA	NA	NA
21–50 mm	1.642	0.746–3.880	0.235	NA	NA	NA
51–100 mm	2.571	1.173–6.054	0.023	NA	NA	NA
> 100 mm	5.521	2.443–13.370	< 0.001	NA	NA	NA
Unknown	4.376	1.897–10.795	< 0.001	NA	NA	NA
*Histological grade (%)*						
Grade I–II	Ref			Ref		
Grade III–IV	1.754	1.079–2.869	0.024	2.178	1.703–2.792	< 0.001
Unknown	0.826	0.590–1.156	0.266	1.400	1.176–1.666	< 0.001
*AFP (%)*						
Negative	Ref			Ref		
Positive	1.796	1.191–2.728	0.006	1.462	1.194–1.791	< 0.001
Unknown	1.387	0.844–2.289	0.198	1.240	0.984–1.563	0.068
*Fibrosis scores (%)*						
Ishak 0–4	Ref			Ref		
Ishak 5–6	2.528	1.174–5.650	0.020	1.073	0.735–1.566	0.715
Unknown	2.043	0.993–4.369	0.058	1.244	0.878–1.762	0.218
*T stage (%)*						
T1–2	Ref			Ref		
T3–4	2.061	1.501–2.838	< 0.001	1.363	1.171–1.588	< 0.001
TX	1.714	0.977–3.022	0.061	1.249	1.030–1.516	0.024
*N stage (%)*						
N0	NA	NA	NA	Ref		
N1	NA	NA	NA	1.210	1.029–1.426	0.022
NX	NA	NA	NA	0.994	0.821–1.206	0.954
*Bone metastasis (%)*						
No	NA	NA	NA	Ref		
Yes	NA	NA	NA	1.239	1.050–1.463	0.011
Unknown	NA	NA	NA	0.715	0.487–1.057	0.089
*Lung metastasis (%)*						
No	NA	NA	NA	Ref		
Yes	NA	NA	NA	2.195	1.901–2.537	< 0.001
Unknown	NA	NA	NA	1.214	0.839–1.763	0.304
*Surgery (%)*						
No	Ref			Ref		
Local tumor destruction	0.187	0.053–0.502	0.003	0.139	0.067–0.265	< 0.001
Wedge resection	0.083	0.012–0.332	0.002	0.204	0.089–0.441	< 0.001
Lobectomy	0.020	0.001–0.108	< 0.001	0.077	0.032–0.165	< 0.001
Surgery but the specific operation unknown	NA	NA	NA	0.649	0.141–2.597	0.554
*Radiotherapy (%)*						
No/Unknown	Ref			Ref		
Yes	0.080	0.041–0.145	< 0.001	0.342	0.281–0.415	< 0.001
*Chemotherapy (%)*						
No/Unknown	Ref			Ref		
Yes	0.144	0.109–0.190	< 0.001	0.163	0.142–0.187	< 0.001

*AJCC* American Joint Commission on Cancer, *HCC* hepatocellular carcinoma, *CI* confidence interval

### Nomogram construction

**Fig. 2:**
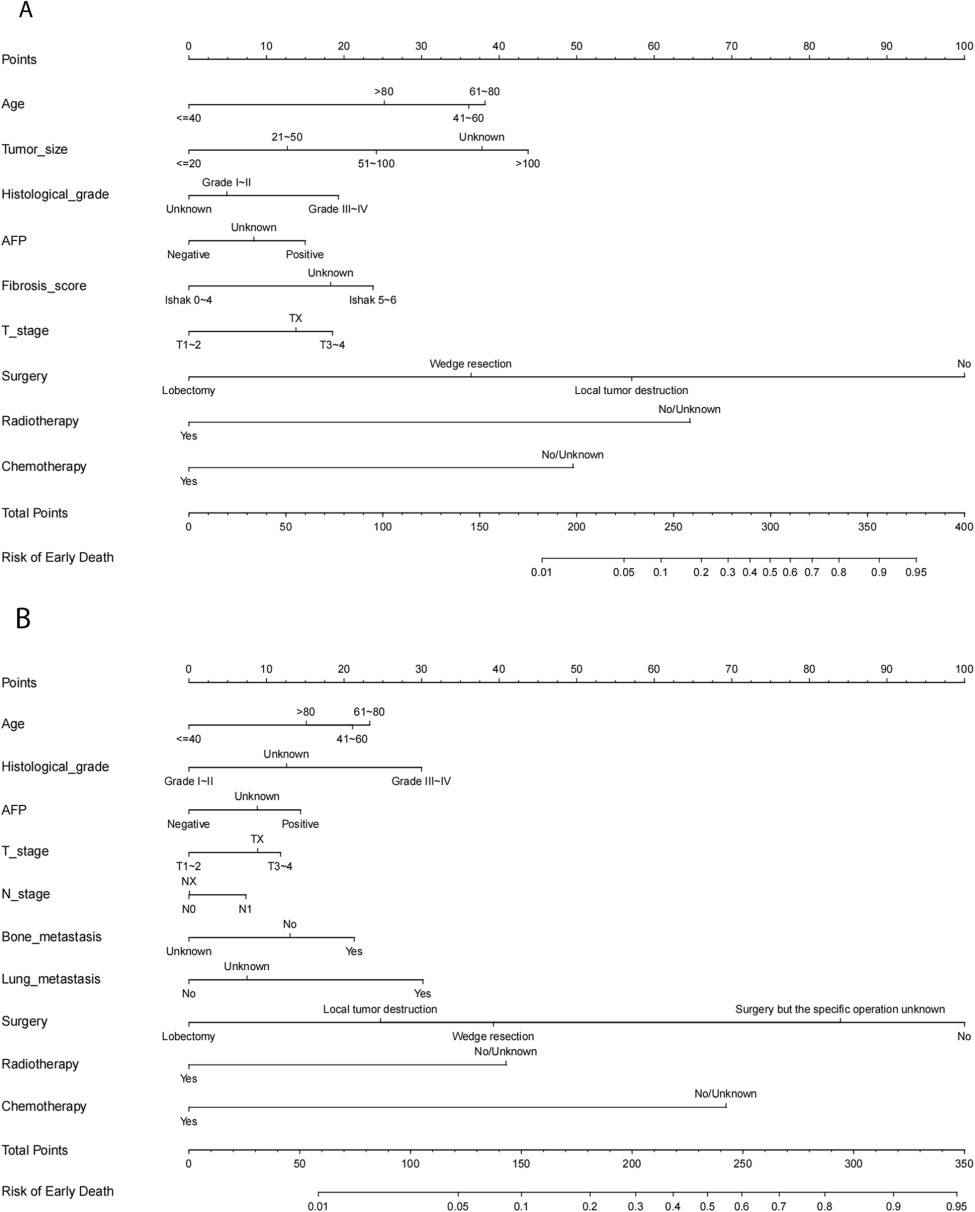
**A, B** The nomograms of early death in patients with advanced HCC. **A** stage IVA, enrolling in 9 risk factors; **B** stage IVB, enrolling in 10 risk factors. Abbreviations: alpha-fetoprotein

Based on the independent and significant risk factors identified by multivariable logistic regression analysis, independent predictive models were developed to predict the probability of early death in patients with advanced HCC, which were presented as nomograms (Fig. 2A, B). In the nomograms, the total points could be obtained by adding up the points for each risk factor, and then the probability of early death could be estimated. For example, a 70-year-old stage IVB patient with lung metastasis of HCC, histological grade III, AFP positive, and only receiving chemotherapy, had an early death probability of about 50%.

### Performance and validation of nomograms

**Fig. 3:**
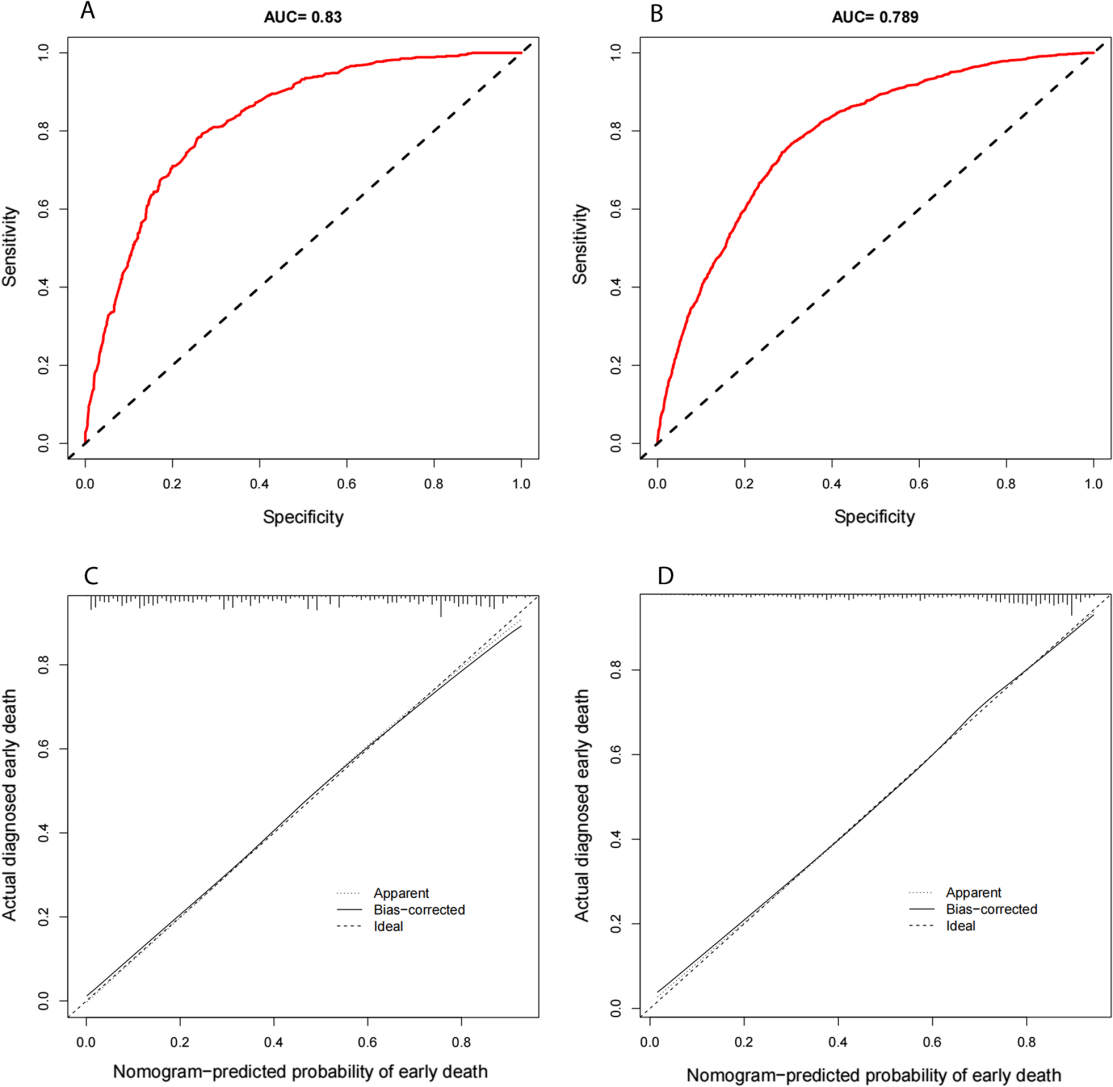
**A, B** The receiver operating characteristic (ROC) curves for the nomogram. **A** stage IVA; **B** stage IVB. **C, D** The calibration curves plots for the nomogram (bootstrapping, 1000 re-samplings). **C** stage IVA; **D** stage IVB. Abbreviations: AUC, area under the curve

**Fig. 4:**
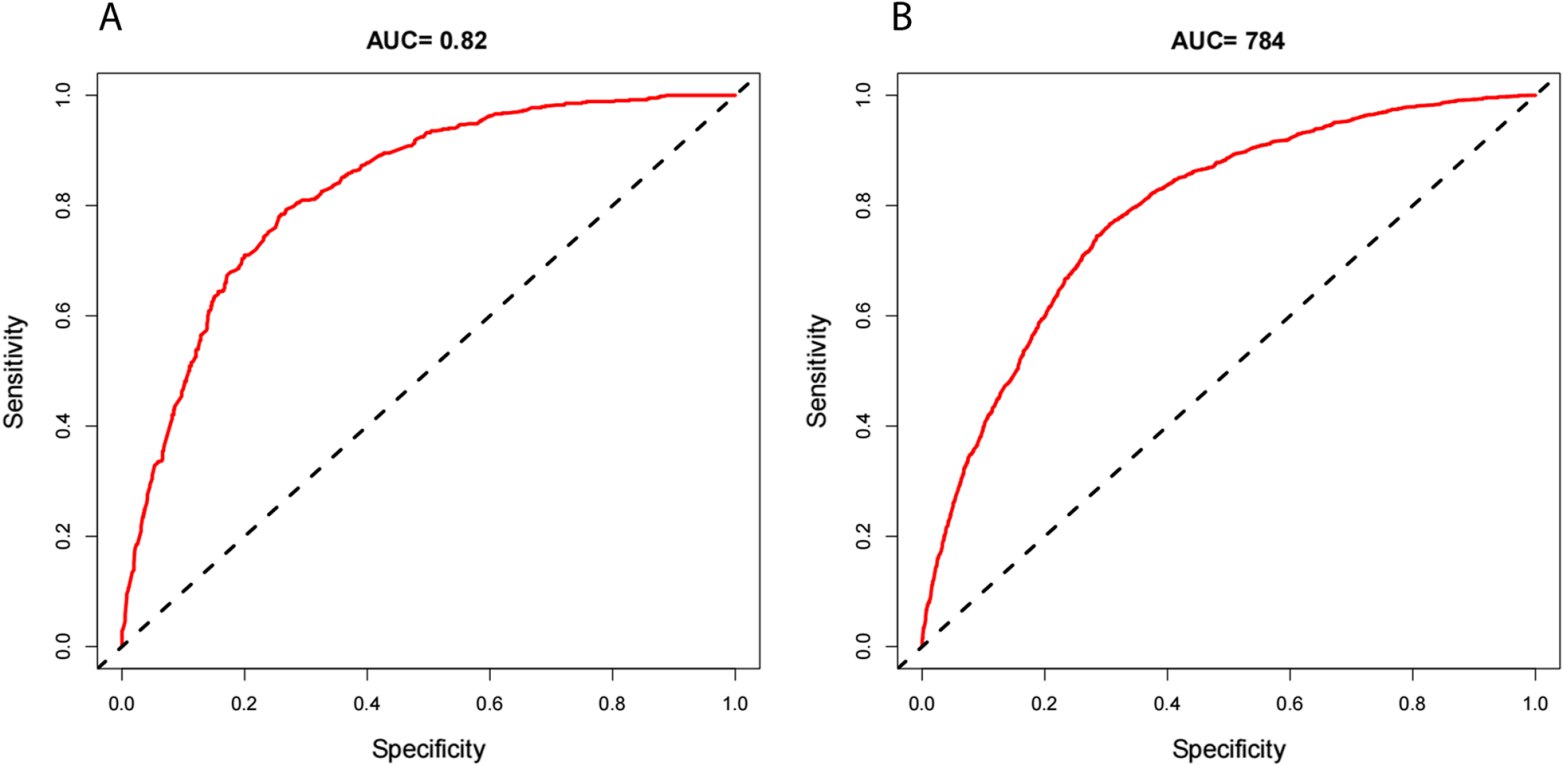
**A, B** The ROC curves after cross-validation (k = 10) for the nomogram. **A** stage IVA; **B** stage IVB. Abbreviations: AUC, area under the curve

Figure 3A, B show the ROC curves of the nomogram for predicting early death in stage IVA and stage IVB HCC patients. In stage IVA patients, the area under the ROC curve (AUC) was 0.830 (95% CI 0.809–0.851); and in stage IVB patients, the ROC curve (AUC) was 0.789 (95% CI 0.768–0.810), indicating that the nomograms had significant predictive ability. Calibration curves were used to evaluate the true compliance of the model. The abscissa was the nomogram-predicted probability of early death, and the ordinate was the actual diagnosed early death, ranging from 0 to 1. The dotted line connecting the two opposite corners was the ideal reference line, and the closer the remaining curves are to the line, the closer the predicted value was to the actual value. In the present study, all calibration curves were close to the diagonals line, indicating a good agreement between the actual observations and predictions (Fig. 3C, D). Internal verification was conducted through bootstrapping (1000 re-samplings) and cross-validation (k = 10). The *C*-indexes after bootstrapping were 0.820 (95% CI 0.799–0.841) and 0.785 (95% CI 0.764–0.0.806) in stage IVA and stage IVB, respectively. Figure 4A, B show the AUCs after cross-validation (k = 10), which were 0.820 (95% CI 0.799–0.841) and 0.784 (95% CI 0.763–0.805) in stage IVA and IVB, respectively, suggesting that the nomograms have good prediction performance.

### Clinical utility

**Fig. 5:**
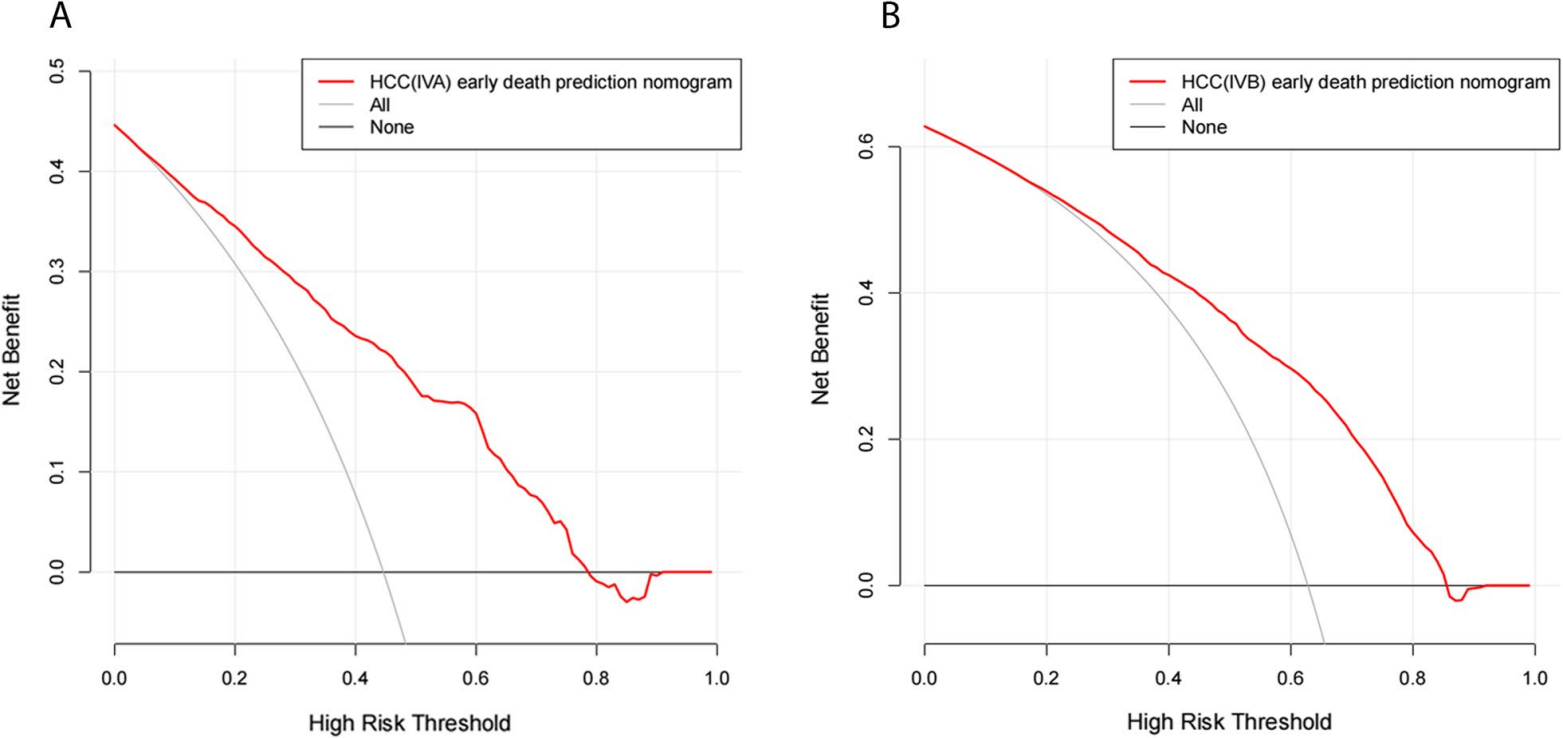
**A, B** The decision curve analyses (DCA) for the nomogram. **A** stage IVA; **B** stage IVB. Abbreviations: HCC, hepatocellular carcinoma

DCA was used to evaluate the clinical applicability of the nomograms. Figure 5A, B show that, in stage IVA patients, the threshold probability was 1–78%; and in stage IVB patients, the threshold probability was 1–85%. Therefore, the constructed nomograms could well assist clinicians in accurately evaluating early death of patients with advanced HCC.

## Discussion

At present, most existing studies have been based on the exploration of early hepatocarcinoma and the long-term survival rate of patients. As an example, Llovet et al. reported that although treatment techniques and strategies in recent years have markedly improved, the 1-year, 2-year, and 3-year overall survival rates in advanced HCC were 29%, 16%, and 8%, respectively [11]. As such, the long-term survival rate of patients with advanced liver cancer is significantly low, and most patients will experience early death. So far, there is a scarcity of research on the early death of patients with advanced HCC. Therefore, developing a series of prediction tools to identify the risk factors and predict the probability of early death to guide clinical treatment is of considerable significance.

The Surveillance, Epidemiology, and End Results (SEER) database (https://seer.cancer.gov/data/), supported by the Surveillance Research Program (SRP) in NCI's Division of Cancer Control and Population Sciences (DCCPS), is one of the most representative broad-scale tumor registration databases, and has records of 34.6% of the US cancer registry population [21]. SEER database provides a large amount of evidence-based medical information, including patients' general information (such as gender, age, race, and marital status), as well as tumor size, TNM stage, histological grade, survival time, and vital status, which contribute to clinical medical research and evidence-based medical practice.

Although the AJCC staging system has been commonly used in the prognosis assessment of primary liver cancer, there are certain limitations in that crucial risk factors such as age, gender, race, histological grade, and treatment solutions are not included. The innovation of the present research lies in the first-time development of nomograms for early death of advanced HCC, which include the aforementioned risk factors.

Nomograms are straightforward and accessible tools, and are the visualization result of various regression analysis. Notably, nomograms have been widely used in malignant tumor risk and prognosis assessment in recent years [22]. The present nomograms were based on the SEER database, which have the characteristics of a large sample size and complete patient follow-up information. Therefore, the nomograms are more exact and stable [23, 24]. The AUC of the present nomograms was found to be greater than 0.75, indicating significantly high accuracy. Additionally, the results of internal validation also illustrate that the nomograms had good predictive ability and calibration ability. With the help of nomograms, clinicians can add the scores corresponding to each risk factor to obtain a total score, so as to accurately predict the probability of early death in advanced HCC patients, and then implement targeted therapy as soon as possible.

Wan et al. [25] constructed a prognostic scoring model for the long-term survival rate of elderly liver cancer patients based on the SEER database, but did not develop a nomogram; and Liu et al. [26] established a prognostic nomogram model for hepatocellular carcinoma based on the SEER database, but did not evaluate the clinical net benefit rate of the nomogram. Traditional ROC curve analysis is merely a statistical abstraction and cannot provide the direct clinical value [27], whereas DCA is a significant analysis in assessing whether a predictive model can be used in clinical practice and whether patients can benefit. Despite the benefits thereof, few studies have adopted the DCA approach in assessing the net benefit of predictive models. In the present study, the DCA results show that, when the threshold probability was between 1 and 78% in stage IVA patients and 1% and 85% in stage IVB patients, the net benefit of the nomogram was better than that in all-patient-death or no-patient-death scenarios.

In the present study, regardless of stage IVA or stage IVB patients, age was significantly associated with early death, which is consistent with the results of previous studies [28, 29]. From the SEER database data and the existing research, HCC is obviously more likely to occur in males [30]; however, gender was not a risk factor for early death of advanced HCC patients. In stage IVA patients, the probabilities of early death in male and female patients were 45% and 42%, and those of male and female patients with stage IVB were 63% and 64%, respectively. Further, the results show that race and marital status were not risk factors for early death of patients. Except for the aforementioned demographic characteristics, the results also show that in patients with stage IVA, larger tumor size, higher histological grade, AFP positive, higher fibrosis scores, T3–4 stage, and patients who had not received surgery, radiotherapy, or chemotherapy were at higher risk of early death, which is consistent with the previous findings of Zhang et al. [31]. In patients with stage IVB, higher histological grade, AFP positive, T3–4 stage, N1 stage, bone metastasis, lung metastasis, and those without surgery, radiotherapy, or chemotherapy, there was a tendency to experience an early death, which is consistent with the previous findings of Zhang and Chen et al. [32, 33].

As previously reported, tumor size is an independent risk factor for HCC recurrence and death after HCC resection. Taking 5 cm as the boundary, the 5-year recurrence-free survival (RFS) and overall survival (OS) rates in the ≤ 5 cm group were 38.3% and 61.5%, while those in the > 5 cm group were 25.1% and 59.9% [34]. However, the results of the present study show that tumor size was not a risk factor for early death in patients with stage IVB, which could be attributed to stage IVB HCC being mostly caused by the recurrence of early liver cancer after surgery. Here, the disease progressed rapidly, and the tumor size may have had less impact on the early death of patients. The results also illustrate that, in stage IVB patients, the fibrosis score was also not a risk factor for early death. Liver fibrosis is a chronic inflammatory process, and has been reported to have no effect on OS and RFS until developing into liver cirrhosis [35]. However, stage IVB HCC had the characteristics of rapid progression and extensive invasiveness, and cirrhosis had no chance to develop, which might be why the fibrosis score was not a risk factor for early death in patients with stage IVB [36]. The brain is one of the most likely sites for metastasis in patients with advanced HCC [32, 37, 38], but the present results did not show that brain metastasis was significantly associated with the early death of patients. Such findings could be attributed to the small number of occurrences of brain metastasis in liver cancer and the insufficient sample size.

At present, liver cancer has entered a multimodal diagnosis and treatment era. In addition to the commonly used methods of ultrasound, magnetic resonance imaging, and computed tomography, tumor markers are also emerging as a significant factor in the diagnosis of liver cancer [39]. Tumor markers that contribute to the diagnosis of HCC include AFP heterogeneity, Glypican-3, osteopontin, Des-γ-carboxyprothrombin, Golgi protein-73, abnormal pro-thrombin, and heat shock protein [40]. Among said markers, AFP is the most widely accepted serum biomarker for the diagnosis of HCC; however, the specificity and sensitivity were found to be 72–90% and 39–65%, respectively. Moreover, the early diagnosis efficiency of AFP was only 9–32%, and cholangiocarcinoma did not express AFP, which limited the clinical use thereof [41, 42]. As such, if said tumor markers can be combined with epidemiology and clinical pathology, a more accurate prediction model could be established to guide the individualized treatment of HCC.

Sorafenib is currently considered to be the standard frontline therapy for advanced HCC. However, a large number of adverse events mainly including gastrointestinal or skin diseases have been found in patients taking sorafenib. In severe cases, sorafenib can cause high blood pressure and abdominal pain, leading to interruption of treatment [43]. Approximately 30% of patients with advanced HCC can benefit from sorafenib, and such patients will usually develop drug resistance within 6 months [44]. Therefore, for the treatment of advanced HCC, new drugs need to be explored and the process of tumor resistance needs to be further understood.

Whether HCC patients with lymph node invasion should accept surgery treatment remains a controversial issue. However, in the present study, the outcome indicates that surgery had an important effect on the improvement of early death in advanced HCC. Moreover, previous studies have stressed that surgery had beneficial value for advanced HCC patients, especially for those with regional lymph node invasion [45]. As such, although the guidelines recommend targeted therapy as the frontline treatment for advanced HCC, such therapy might be more suitable for patients with stage IVB. For patients with stage IVA who only have regional lymph node metastasis, surgery might also be a better treatment option. Despite such recommendations, in consideration of the small number of patients undergoing surgery in the present study, a more prudent approach would be to set strict indications for surgery in advanced HCC based on the clinical conditions of the patients. Further, large-scale prospective studies are required to verify the surgical value of advanced HCC.

Inevitably, there are several limitations in the present study. First, although the SEER database provides a large enough sample size, there is still a lack of several potential risk factors, which may be related to early death, such as the specific location of regional lymph node metastasis, the patients' past medical history, adverse habits (drinking and smoking history), postoperative tumor remnants, the specific methods of radiotherapy and chemotherapy, and other tumor markers aside from AFP. Second, the SEER database has limitations in HCC tumor staging. Specifically, the AJCC staging system lacks important prognostic information, including Child–Pugh classification and the patients' performance status, and thus, cannot be widely endorsed for HCC. There is a possibility that the patients in the present research may have included Child–Pugh class C, who were in Barcelona Clinic Liver Cancer (BCLC) stage D, and surgery, radiotherapy, and chemotherapy were not recommended. Third, as a retrospective study, selection bias caused by censoring data was unavoidable. Finally, although internal verification suggests that the nomograms had good predictive capabilities, multiple centers and large sample size data are required for external verification to avoid overfitting.

## Conclusion

In conclusion, based on the large sample size provided by the SEER database, the risk factors for early death of patients with advanced HCC were identified and nomograms were developed. The results of internal verification suggest that the nomograms had significantly high accuracy. The nomograms may help oncologists and clinicians identify risk factors and probability of early death more quickly and accurately, so as to allow for more precise individualized treatment plans to be formulated, thereby improving the patients' survival probability and quality of life.

## Supplementary Information

**Additional file 1: Table S1**. A dataset of the information of stage IVA HCC patients.

**Additional file 2: Table S2**. A dataset of the information of stage IVB HCC patients.

## Data Availability

The original data for this study is obtained from the SEER database. The detailed website can be found at: https://seer.cancer.gov/data/. More specific data used in this study are available from the authors upon reasonable request and with permission of the SEER database.

## Abbreviations

HCC: Hepatocellular carcinoma
SEER: Surveillance, epidemiology, and end results
C-index: Concordance index
ROC: Receiver operating characteristic
AUC: Area under the curve
DCA: Decision curve analysis
TACE: Transcatheter arterial chemoembolization
SRP: Surveillance research program
NCI: National Cancer Institute
DCCPS: Division of cancer control and population sciences
ICD-O-3: International classification of tumor diseases third edition
OR: Odds ratio
CI: Confidence interval
CSS: Cancer-specific survival
OS: Overall survival
RFS: Recurrence-free survival
AFP: Alpha-fetoprotein
AJCC: American Joint Commission on Cancer
BCLC: Barcelona Clinic Liver Cancer
